# Simulation and Printing of Microdroplets Using Straight Electrode-Based Electrohydrodynamic Jet for Flexible Substrate

**DOI:** 10.3390/mi13101727

**Published:** 2022-10-12

**Authors:** Dazhi Wang, Zeshan Abbas, Liangkun Lu, Chang Liu, Jie Zhang, Changchang Pu, Yikang Li, Penghe Yin, Xi Zhang, Junsheng Liang

**Affiliations:** 1 Key Laboratory for Micro/Nano Technology and System of Liaoning Province, Dalian University of Technology, Dalian 116024, China; 2 Key Laboratory for Precision and Non-Traditional Machining Technology of Ministry of Education, Dalian University of Technology, Dalian 116024, China; 3 Ningbo Institute of Dalian University of Technology, Ningbo 315000, China

**Keywords:** phase-field method, e-jet printing, droplet generation, straight electrode, flexible PET substrate

## Abstract

Electrohydrodynamic jet (e-jet) printing is a modern and decent fabrication method widely used to print high-resolution versatile microstructures with features down to 10 μm. It is currently difficult to break nanoscale resolution (<100 nm) due to limitations of fluid properties, voltage variations, and needle shapes. This paper presents developments in drop-on-demand e-jet printing based on a phase-field method using a novel combined needle and straight electrode to print on a flexible PET substrate. Initially, the simulation was performed to form a stable cone jet by coupling an innovative straight electrode parallel to a combined needle that directs the generation of droplets at optimized parameters, such as *f* = 8.6 × 10^−10^ m^3^s^−1^, Vn = 9.0 kV, and Vs = 4.5 kV. Subsequently, printing experiments were performed using optimized processing parameters and all similar simulation conditions. Microdroplets smaller than 13 μm were directly printed on PET substrate. The model is considered unique and powerful for printing versatile microstructures on polymeric substrates. The presented method is useful for MEMS technology to fabricate various devices, such as accelerometers, smartphones, gyroscopes, sensors, and actuators.

## 1. Introduction

Flexible electronics is a technology that allows the construction of electronic circuits on flexible substrates (i.e., PET and PEN), thus making them flexible and stretchable [1]. It enables new product paradigms that are not possible to build with conventional semiconductors and glass substrates. Flexible printed electronics, also known as printable circuits, are used in electronic devices due to their flexible specifications [2]. Industrial and medical equipment that need numerous small interconnections often use flexible circuits. Another popular example of versatile circuits is mobile phones [3,4]. For satellite power, adjustable solar cells have been created extensively. These cells are an excellent fit for the application since they are lightweight, rapidly deployable, and can be rolled up for launch [5]. Additionally, they may be sewed into jackets and bags. Flexible substrates provide a number of benefits over rigid glass substrates, including less weight and simpler roll-up storage [6]. However, in the sphere of electronics manufacturing, PET substrates are frequently recognized as a highly insulating material. Due to its nonconductive qualities, it cannot be employed as a direct electrode in the e-jet printing process, and the stability of the cone-jet formation is greatly reduced [7]. E-jet printing, which allows for the manufacture of micro/nanostructures, has recently attracted a lot of attention because of its usefulness in flexible printed electronics [8], micro/nanoscale 3D printing [9], research in optoelectronic displays [10], and further in thin-film transistors (TFTs) [11]. In comparison to slot-die coating and inkjet printing techniques, e-jet printing techniques offer a number of benefits, including three-dimensional structuring, multispace-selective modeling, high processing distribution efficiency, high throughput, and a contactless approach [12].

The most popular printing method in micro/nanodevices is inkjet printing, in which the functional ink is supplied to a substrate surface using a range of techniques, such as heat bubbles or piezoelectric forces [9]. Park et al. used double-sized nozzle diameters and determined the resolution of microstructures on substrate using inkjet printing. It has been found that this can result in throat blockage when a small droplet is fabricated using highly functional viscous ink. On the other hand, when using an e-jet printer, a droplet that is considerably smaller than the nozzle diameter may be created. This is due to the strong electrostatic forces that the applied voltage creates that overpowers the surface tension force and drives the functional ink toward the substrate surface [6]. E-jet atomization has lately gained attention for printing micro/nanosized designs since it may be employed on a variety of materials, including metals, organics, and even biological components, without causing heat damage [13]. In contrast to more complex varieties, this technology operates more effectively with the simplest nozzle construction [14]. In e-jet atomization, charged fluid is extracted from the nozzle when the electrical force locally exceeds the functional surface tension of the ink [4]. As a result, the e-jet printing method, which is regarded as the most useful tool in micro- and nanofabrication, significantly improves the resolution of microstructures [15] for flexible printed electronics.

Many studies have introduced phase-field methods similar to what we are presenting in this work. Exploratory use was made of electrodes and needle combinations to explore controlled and stable cone jets in order to print droplets on a flexible substrate. A numerical modeling of e-jet and printing microstructures on a flexible substrate was also carried out by Abbas et al. They identified the best settings for creating a drop-on-demand (DoD) e-jet to print stable microstructures and control them on a flexible insulating substrate [1]. Another model developed by Abbas et al. included printed direct writing structures on flexible substrates and a computer simulation of robust cone-jet production [7]. The work provided a two-phase-field approach using a series of copper control electrodes to tune settings for stable cone-jet morphology on a PET substrate. Lee et al. provided a simulation model that addressed satellite suppression and the influence of residual charges stored on an insulating substrate. In order to maintain its strength, the jetting route relied on field vectors around it to converge on the target and concentrate their strong spots along the line region [16]. Hartman et al. introduced an e-jet breakup model in order to develop e-jet atomization during the cone-jet mode formation. The study acknowledged the electric field around the Taylor cone. Likewise, it was estimated that the surface charge density at the fluid surface was grounded [17]. Wei and Dong constructed a computational fluid dynamics (CFD) simulation model to solve the electric forces and surface tension forces for the high resolution of microdroplets on a glass substrate using a DoD technique [18]. Kim et al. presented inkjet printing of silver conductive tracks on flexible substrates. Nanosized silver particles of 21 nm diameter were used for direct metal printing [19,20,21]. Singh et al. introduced an e-jet phase-field simulation model for printing of microscopic nanoconstructs. They examined the effect of different parameters on electrojet ejections and effectively guided the design of printheads for e-jet printing systems [22]. Pan and Zeng used e-jet printing technology to create polyethylene oxide helical fibers on a PET substrate. Additionally, copper electrodes and a stainless steel nozzle were used to produce a regulated cone jet on a flexible PET substrate, although the resulting morphology was hopping and bending towards the substrate surface [23]. However, the primary factor in the failure of the consistency in cone-jet profile to print on the PET substrate. It was influenced due to the repulsive forces originated from the electrical charges that remained on the substrate [24]. The properties of viscoelasticity in cone-jet mode were investigated by Panahi et al. Their numerical simulation employed the phase-to-phase approach, and model equations were solved to produce viscoelastic jets, such as the conical plane and adhesive plane [25]. A numerical simulation of cone-jet production in ring electrode-based e-jet atomization was suggested by Lim et al. The study’s goal was to produce monodisperse droplets on the substrate’s surface using a CFD model [26]. Jaworek et al. carried out an experimental investigation to identify several spraying modes using various voltage and flow velocity values. The need for long-term adjustments for the cone jet was then shown by tailoring a set of applicable parameters [27]. According to the recommended literature, using focused electrodes and a pulse voltage at the needle can advance the e-jet printing process on insulating surfaces; however, issues with residual charge still exist. Thus, a unique geometric model has been studied and introduced by constructing a straight electrode with a combination needle during e-jet printing of PET substrate.

To explore stability in cone-jet morphology for the generation of small droplets, we present a phase-field numerical simulation by introducing a novel straight electrode parallel to the combination needle structure. The procedure is based on a production-oriented technology for the DoD process, in which a straight electrode was constructed to achieve stability and regulate e-jet morphology for printing tiny constructions on flexible PET substrate. Previously, we attempted to regulate cone-jet form and printed microstructures using separate control electrodes surrounding the combination needle. The size of the cone-jet morphology was regulated by changing the needle design. Subsequently, we attempted to reduce the residual charges on the surface of flexible PET substrate by regulating DC positive pulse voltage, which still has challenges and produces distortion among the meniscus profile. In the existing work, electric potential effect was examined in 2D and 3D plot assembly using a novel straight electrode. Thus, the particular diameter of the straight electrode was selected at specific values of voltage and flow rate, which significantly affects the size of the cone jet and droplet production. The simulation generates very smooth and stable droplets, and does not emit residual charges on the PET substrate surface. The aim of this study was to control the cone-jet morphology for production of stable microstructures on flexible PET substrate for flexible printed electronics. This simulation model examines the main parametric impact on the reduction of instability in MEMS devices, especially for the DoD e-jet printing technique.

## 2. Computer Simulation and Experimentation

### 2.1. Materials

Based on the DoD method, simulations and experimental studies are carried out, and a typical UF 3808 curing ink is regulated in a combined needle. The flexible substrate (e.g., PET) is used in its natural state due to its physical properties as a colorless and semicrystalline resin. Depending on its surface energy, PET can be semisolid to solid and is a very light polymer material. The physical properties of the curing ink (Henkel Corporations Group Ltd., Stamford, CT, USA) were obtained from the suppliers. The polymer material can provide excellent mechanical properties for electronic device application. These materials are also widely used in flexible printed electronics due to their high surface energy. Likewise, flexible PET substrates are broadly used in biotechnology and electrical equipment, and have a high-strength barrier that can resist interference from various chemical characteristics. The steel needle and straight electrode (copper) were used in the experiments according to the simulation results. The straight electrode was fixed around the combined needle using the insulating PMMA holder. The thickness of the PET substrate was determined to be 0.5 mm in the simulation and was also designed in the experiments. Table 1 describes the physical properties of the ink used for the simulations to produce stable microdroplets.

### 2.2. Formulations for Fluid Flow

**Figure 1:**
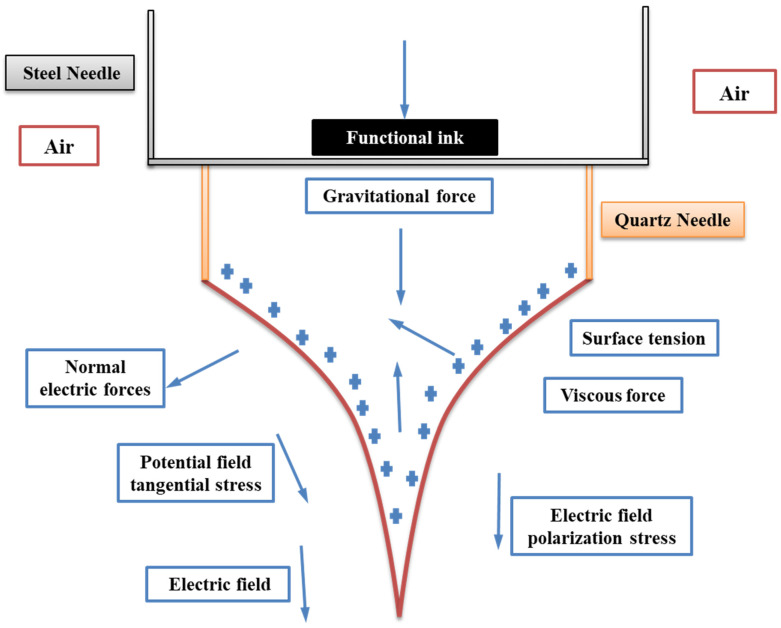
Influence of different forces around the Taylor cone during DoD e-jet printing method.

The Navier–Stokes equations and the principle of conservation of mass are taken into consideration as the foundation for the fluid flow motion calculated in COMSOL Multiphysics (COMSOL Inc., Stockholm, Sweden, China Brach: Comus Software Technology (Shanghai) Co., Ltd) and as described in Equation (1). In this work, the theory of e-jet printing is discussed. The fluid flow, applied electric field, and Cahn–Hilliard equations are used to simulate the cone-jet shape. The modeling of DoD e-jet printing, which is based on the phase-field technique, entails tracing the functional liquid–gas interface at the needle apex. Figure 1 shows a schematic representation of the distribution of forces influencing cone-jet morphology. The two phase fields of the inner liquid and the air in this simulation study were considered to be immiscible and incompressible.

(1) $$\frac{\delta \rho }{\delta t}\;\cdot \;(\nabla \cdot \vec{\mu )}=0$$

where $\vec{\mu }$ signifies the flow rate of the fluid. The Taylor–Melcher leaky dielectric model [28,29] served as the foundation for the various forces acting on the interface of a functional fluid. In the numerical simulation model, the Navier–Stokes incompressible flow equation is utilized to figure out laminar fluid flow, which is given by Equation (2).

(2) $$\rho \frac{\partial \vec{u}}{\partial t}+\rho \vec{(u}\cdot \nabla )\cdot \vec{u}=\nabla \cdot \left[-\mathrm{pl}+\mu (\nabla \vec{u}+{\left(\nabla \vec{u}\right)}^{T}\right]+F_{\mathrm{st}}+F_{\mathrm{es}}+\rho g$$

The liquid density is *ρ*, the gravitational acceleration is *g*, and the surface tension and electric forces are denoted by *F*_st_ and *F*_es_, accordingly. Consequently, λ is regarded as the actual distance between the functional liquids, which is employed as a variable, and is also known as the determinant coefficient, as stated in Equation (3).

(3) $$\frac{\partial \varphi_{i}}{\partial t}-\nabla \cdot \vec{(\mu }\varphi_{i})=(\nabla \cdot \frac{\gamma \lambda }{\sum_{T}})\cdot \Delta \psi ,\varphi =phipf\;\lambda =\frac{3\varepsilon phi\sigma }{\sqrt{8}},\gamma =Χ$$

(4) $$\left\{\begin{array}{l} \frac{\partial \varphi_{i}}{\partial t}-\nabla \cdot (\vec{\mu }\varphi_{i})=(\nabla \cdot \frac{{Μ}_{o}}{\sum_{T}})\cdot \Delta \eta_{i}\\ \eta_{i}=\frac{4\sum_{T}}{\theta }\sum_{i}\prod i\mp j-[\frac{1}{\sum_{j}}\left(\sigma_{i}F\left(\varphi \right)-\sigma_{j}F\left(\varphi \right)\right)]-(\frac{3}{4}\varepsilon c\Delta \theta \psi ) \end{array}\right.\;i=A,B,C$$

where the surface tension force is expressed as *σ*. In the same way, *σ* and *ε* are computed separately when using Cahn–Hilliard equations [30]. As a result, γ=Χ indicates the driving force of liquids employed as a variable. To control the fluid flow as specified in Equation (4), the concentration of the two liquid phases during the numerical simulation is crucial. For the mass flow of liquids, the Cahn–Hilliard potential is an auxiliary variable that is employed in the variable contour to help with the particle directions described in Equation (5).

(5) $$F\left(\varphi \right)=\sigma_{AB}\varphi_{A}{}^{2}\varphi_{B}{}^{2}+\sigma_{AC}\varphi_{A}{}^{2}\varphi_{C}{}^{2}+\sigma_{BC}\varphi_{B}{}^{2}\varphi_{C}{}^{2}+\varphi_{A}\varphi_{B}\left(\Sigma_{A}\varphi_{A}+\Sigma_{B}\varphi_{B}\right)\sum_{i}=\sigma_{i,j}+\sigma_{i,k}$$

where the two-phase flow model’s phase variables *A* and *B* are utilized in simulation work to specify where each phase is located. Consequently, $\sum_{i}$ signifies the whole impact of surface tension. It is anticipated that the total of the entire two phases with high concentration values will equal one in each numerical box, as stated in Equation (6).

(6) $$\varphi_{A}+\varphi_{B}=1\;\frac{2}{\sum_{i}}=\frac{1}{\sum_{A}}+\frac{1}{\sum_{B}}$$

### 2.3. Formulations for Electric Field

The electric field creates an effective disruption at the needle outlet during the phase field simulation. As a result, the Navier–Stokes equation [31] develops electrical forces and surface tension as body forces in the simulation model described in Equations (7) and (8).

(7) $${\vec{F}}_{\mathrm{es}}=q\vec{E}-\frac{1}{2}{(E}^{2}\nabla \varepsilon )+\nabla \varepsilon_{0}$$

(8) $${\vec{F}}_{\mathrm{st}}=-G\left(\nabla \varepsilon \varphi \right)$$

where ϕ represents the phase-field symbolic variables and *G* is a chemical potential [32]. The chemical potential *G* is explicitly provided in Equation (9).

(9) $$G=\lambda \left[-\left(\nabla^{2}\cdot \varphi \right)+\frac{\varphi \cdot \left(\varphi -1\right)}{\theta^{2}})\right]=\frac{\delta \lambda }{{\delta \theta }^{2}}\left(\psi \phi \right)$$

In the simulation model, where λ is the fluid’s combined energy density, δ a metal needle’s width is often usually referred to as the interface thickness. The ε indicates the electric permittivity of the functional ink and $\varepsilon_{0}$ specifies the permittivity of vacuum for computing the body forces. ∇ϕ also displays the gradient of the scalar electric potential, while $\vec{E}$ is the electric field close to the combined needle system’s tip. The Coulomb force is created when free charges collide with an interface when a ground electrode is present. In Equation (11), according to the definition of Equation (10), the term $\left\{\vec{E}\varepsilon \right\}$ is a volume charge density, and the electric field is guided by the Poisson and volumetric charge density [33], where $\vec{j}$ is a current density that is shown in Equation (12).

(10) $$[\nabla \cdot \{\vec{E}\varepsilon \}]=\rho_{c}$$

(11) $$\frac{{\delta \rho }_{c}}{\delta t}+\nabla \cdot (\phi \phi \cdot \vec{j})=0$$

(12) $$\vec{j}=\rho_{c}\left(u+\mathrm{KE}\right)=0$$

*K* therefore stands for the functional ink’s electrical conductivity. Additionally, it defines the electric current caused by charge conduction and convection along the cone shape. The modeling of the phase field begins with the lowering of free external energy. In the simulation models [30,34], the Cahn–Hilliard equation was produced over time for two immiscible fluids and is presented in Equation (13). In order to trace the two-phase interfaces during the laminar phase, the conservative phase-field form was utilized to identify the interface between the liquid and gas phases in the simulation model as provided in Equation (14).

(13) $$\frac{\partial c}{\partial t}-\nabla \cdot (M\cdot \nabla \cdot \vec{\mu })=\nabla \cdot (\frac{\omega \lambda }{\delta^{2}}\nabla \psi )$$

(14) $$\psi =-\frac{3}{2}[\sigma \varepsilon \Delta \phi +24\frac{\sigma }{\varepsilon }\phi \left(1-\phi \right)\left(1-2\phi \right)]+(\frac{\delta^{2}}{\lambda })\frac{{\delta f}_{\mathrm{outer}}}{\delta \sigma }$$

where µ is the chemical potential of the mixture and σ indicates the surface tension force at the contact, $\frac{{\delta f}_{\mathrm{outer}}}{\delta \sigma }$ is the liquid’s gradient, and M is its mobility factor. The concentration (φ) changes in the liquid state with a sequence form that has a specific value (φ= +1) and, accordingly, the concentration changes in the gaseous state with a special value (φ= −1) are differentiated. The phase-field variables must maintain the state 0 ≤ ϕ ≤ 1 during the phase-field simulation. During the Cahn–Hilliard potential, the physical parameters, namely, σ and ε, are imposed separately. The functional fluids, namely, liquid and gas, are taken into account as a single efficient unit in order to trace the two-phase interface at the needle apex using the phase-field approach [35]. The significant fluid characteristics are shown in Equation (15).

(15) $$\eta =\eta_{g}[\frac{(1-\varphi )}{2}+\eta_{l}\frac{(1+\varphi )}{2}]$$

where η is a real fluid property, $\eta_{g}$ is the gaseous state property, and $\eta_{l}$ is the functional ink property. The dynamic viscosity and relative permittivity of fluids place strong influences on the phase-field variables (φ).

### 2.4. Process of Numerical Simulation

**Figure 2:**
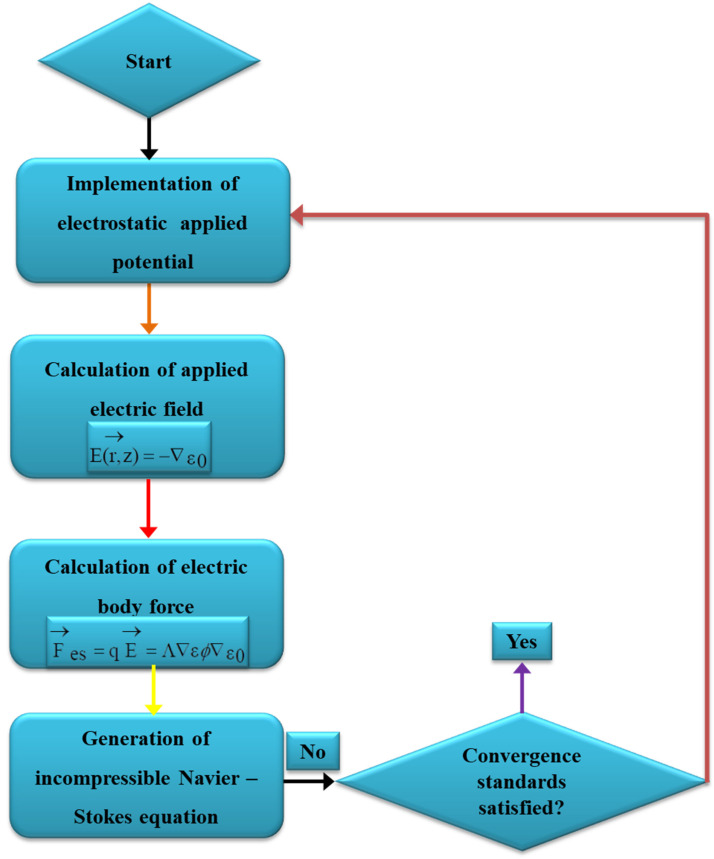
Flowchart of phase-field simulation.

**Figure 3:**
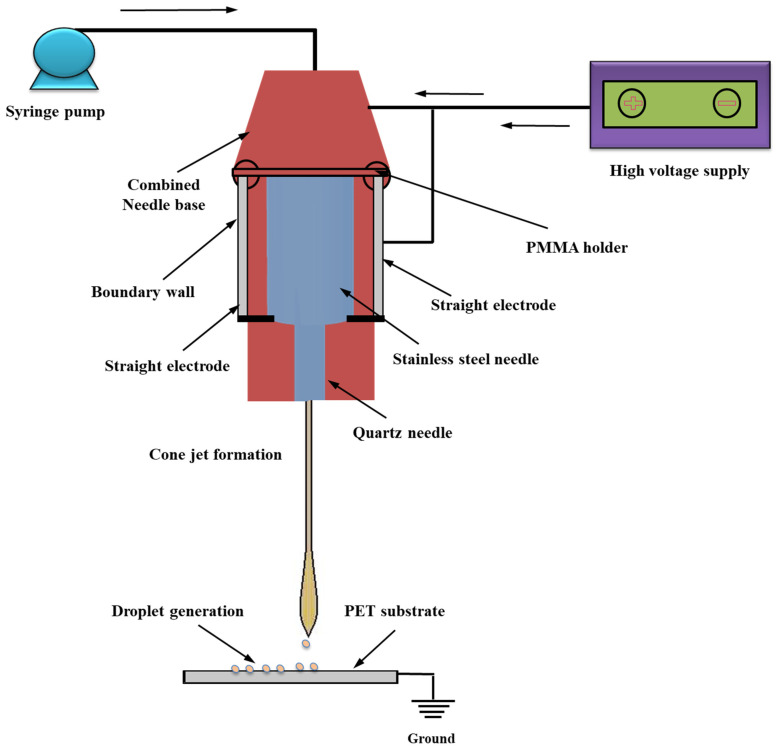
Schematic diagram of DoD e-jet printing method.

An electric field’s expression in a fluid, the monitoring of a two-phase interface, and the coupling of the flow field to the electric field are all described by the equations in the aforementioned sections. In this study, COMSOL Multiphysics software (Version 5.2a) is used to simulate the cone-jet morphology. In this phase-field simulation model, the combined needle system was employed. From Figure 2, it is clear that the cone-jet shape used in e-jet technology includes the linking of two separate physics fields—an electric field and a flow field. An analogous liquid phase and a gas phase are present in the flow field between them. The e-jet Reynolds number states that the laminar flow module is chosen by the flow field since the operating voltage is DC positive pulse voltage [36]. Additionally, the electric field chooses the electrostatic field module, where the Multiphysics module connects the two separate physical fields. The inner liquid–outer air interface’s morphological changes over time are the main focus of the simulation research. As a result, the research kind is transitory. The working electric field is dispersed across the substantial area between the combined nozzle and the ground electrode, in accordance with the theory behind the combined needle e-jet printing procedure. Three-dimensional axial symmetry governs this region, and liquid movement is not taken into account. The axisymmetric electric field in two dimensions may be used to represent the three-dimensional electric field. The diameter of the cone jet is significantly smaller than the inner diameter of the nozzle in order to clearly detect the production of the microdroplets and lower the cost of simulation computation of fluid flow [37]. After the functional fluid is applied, electric field distribution is solely impacted by the flow of liquid near the interface of the e-jet printing nozzle. As a result, the primary simulation domain is restricted to the nozzle’s inner diameter in the phase-field e-jet printing nozzle. The electrostatics and laminar phase-flow physics were computed in Multiphysics contour in accordance with the schematic layout of the phase-field simulation system as shown in Figure 3 to produce stable microdroplets on various insulating surfaces. Basic geometry and boundary conditions from our earlier simulation work for e-jet printing [38,39,40,41] were employed in the axisymmetric model.

#### Establishment of Phase-Field Simulation Model

**Figure 4:**
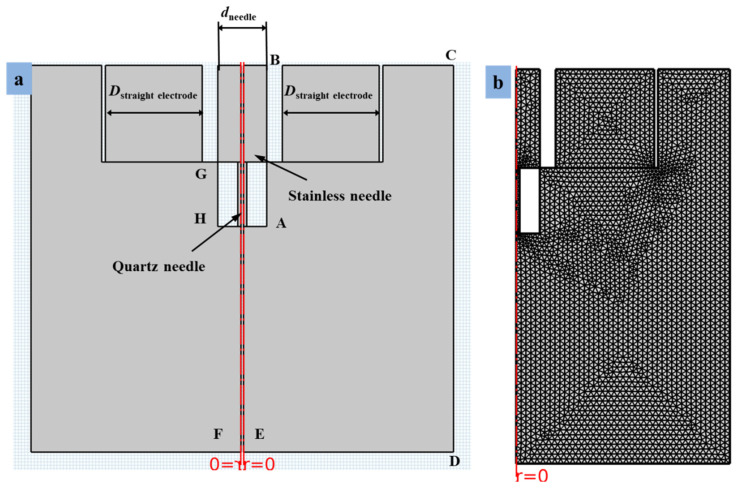
The simulation model for the DoD e-jet printing process. (**a**) Geometry preparations and setting of boundary conditions. (**b**) The finer meshing for the simulation process.

The combined needle e-jet printing process’s axial symmetry is used to transform the three-dimensional structure into a two-dimensional axisymmetric structure, which reduces the amount of calculation and increases simulation efficiency. Figure 4a depicts the geometric model, boundary conditions, and simulation model for e-jet printing. Additionally, the simulation model’s finer meshing system was put up in mesh contour, as illustrated in Figure 4b. The boundary conditions for the simulation of the electrostatic field and flow field are listed in Table 2. Additionally, φ stands for the electrostatic field’s electric potential, and u and P, which stand for fluid velocity and pressure, respectively. A is the inner liquid inlet among them, satisfying the equations *φ* = *V*_0_, *u* = Q_inner_/A_inner_, where V_0_ is the voltage, Q_inner_ is the inner liquid flow rate, and A_inner_ is the inner liquid’s effective flow area. B is the combined needle’s outside wall, satisfying the conditions *φ* = *V*_0_, *u* = Q_outer_/B_outer_, where Q_outer_ is the outer air’s flow rate and B_outer_ is the outer air’s near-wall side effective flow area. The straight electrode’s wall, C, meets the conditions *φ* = *V*_0_ and *u* = 0. D is the straight electrode’s charge density, which meets the conditions *φ* = *V*_0_ and *u* = 0. Additionally, E is the combined needle structure’s exit, satisfying the conditions *p* = 0 and *φ* = 0. G is a geometric model axis-symmetry that meets the conditions *φ*_r =_ 0, u_r_ = 0, where d_r_ denotes the radial component of electric potential and u_r_ denotes the radial component of velocity. F is the border of the complete computational domain, satisfying the conditions *φ* = *V*, *p* = 0, and *V* is a variable value that varies with calculation time. The inner diameter of the steel needle was preserved at 350 μm and the outside diameter was set at 700 μm for the geometric model dimensions that were simulated in this work. Similarly to this, the inner and outer diameters of the quartz capillary were both fixed at 50 μm and 350 μm, respectively. Additionally, the two straight electrodes were employed in tandem with the steel needle in order to produce a steady cone jet for the creation of droplets on insulating surfaces during the e-jet printing process. Each electrode’s width was held constant at 1.8 mm. The distance between the copper straight electrode and needle may also be adjusted, ranging from 0.5 mm to 1 mm. Straight electrode upper coupling was configured with an inner diameter of 580 μm and an outside diameter of 1020 μm. The distance between the needle tip and insulating substrate was kept constant at 200 μm. The following components of the simulation model have been reduced in order to decrease the amount of calculation and increase simulation efficiency and accuracy [42].

(1) The transformation of a three-dimensional model into a two-dimensional axisymmetric model.
(2) Disregard the needle’s outer diameter.

This is due to the fact that the needle’s shape is axisymmetric, and the two-dimensional axisymmetric model can adequately describe the needle model, the simulation process can be substantially streamlined, and the flow field is unaffected by the needle’s outer diameter.

## 3. Results and Discussion

### 3.1. Electric Potential Influence on Cone Jet during 2D Plot Assembly

**Figure 5:**
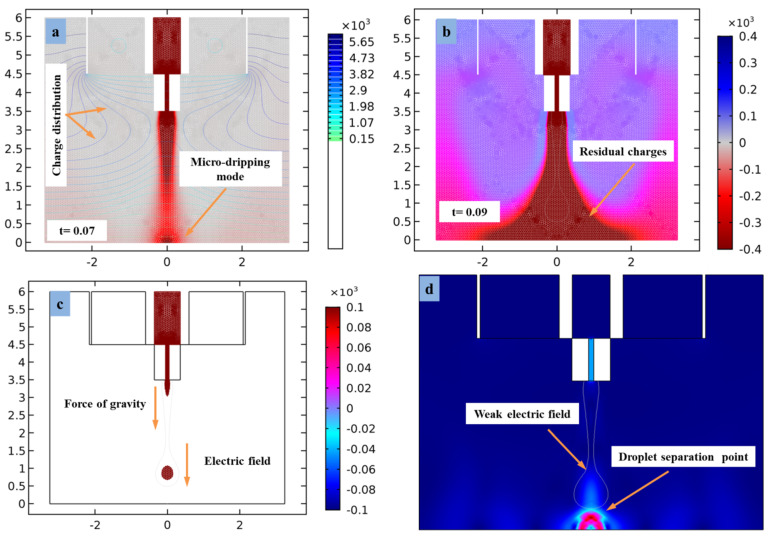
(**a**,**b**) Distribution of volume charge density around cone-jet morphology, (**c**) effect of electric field on droplet, (**d**) inflation in droplet size during weak electrical forces.

During 2D plot assembly, the factors influencing the formation of stable cone-jet morphology are mainly divided into three categories, which are the physical properties of functional liquid (such as dielectric constant, conductivity, viscosity, concentration), process parameters (such as voltage, liquid flow rate, needle straight electrode distance), and external environmental parameters (such as temperature, humidity, airflow speed). Among them, the physical properties determine whether the material is suitable for the e-jet printing process. The process parameters mainly affect the cone-jet morphology and size of the microdroplets. Correspondingly, the external environmental parameters mainly affect the stability and consistency of the cone-jet profile. The simulation study on the formation of the cone-jet morphology shows that the functional solution used in this phase-field method can be very suitable for the e-jet printing process and meets the requirements for the formation of a stable cone jet, as shown in Figure 5a,b. The simulation model in Figure 5a shows that at the time interval (t = 0.07), since the voltage effects are normal, then the charge density distribution lines are very thin around the combined needle. However, the applied voltage, inner liquid flow rate, and needle straight electrode distance affects the shape of the cone jet and diameter of the droplets, and in the results of these parameters, its mechanism of action reveals microdripping printing mode. The charge distribution is the most immediate internal factor in the formation of a cone-jet morphology for the generation of stable microdroplets on the flexible substrate surface.

While the time interval (t = 0.09) increases shown in Figure 5b, the light-blue color indicates the distribution of electrical charges around the needle interfaces and near the needle wall. Similarly, the weak charges become the cause of accumulation of residual charges on the insulating substrate surface. The territory around the needle tip determines that abundant electrical charges produce a satellite effect that disrupts the formation of the cone et near the surface of the flexible PET substrate. In order to solve the problems of microdroplet stability and poor consistency of printing structure, an e-jet printing straight electrode device is developed in this 2D assembly section. The straight electrode is placed directly parallel to the combined needle and forms the required intensity of electric field between the combined needle and ground electrode. Before printing, we set a proper voltage at the straight electrode that overcomes the effect of surface tension at the needle interface by generating a solid electric field effect. At this time, a very thin e-jet film will be formed around the microdroplet, which is on the upper surface of the ground electrode, as shown in Figure 5c. When the pulse voltage exceeds a certain value (approximately V_n_ = 9.2 kV), the Coulomb repulsion between electric charges on the e-jet surface is too large, causing Rayleigh scattering and the microdroplet becomes unstable and begins to convert in multi-jet. Therefore, the printed structures are scattered and disordered. At this time, if the voltage continues to rise, the droplet will be cut off and even an electrical breakdown will occur. Conversely, if the voltage is too low (approximately V_s_ = 3.1 kV), then electric field strength becomes weaker and the electric field force generated by the electric charges accumulated at the two-phase interface is not sufficient to pull the inner liquid. Furthermore, the droplet shape is coarse and unstable, which produces inflation on the substrate surface due to high insulating properties of PET, as shown in Figure 5d. Thus, when the droplet reaches the outlet of the substrate, it flows to the ground electrode and the stability of droplet at the outlet of PET substrate decreases due to the charge density drops sharply with accumulation of residual charges. Units on the x–y axis for Figure 5 are in millimeters.

### 3.2. Electric Potential Evaluation during 3D Plot Assembly

**Figure 6:**
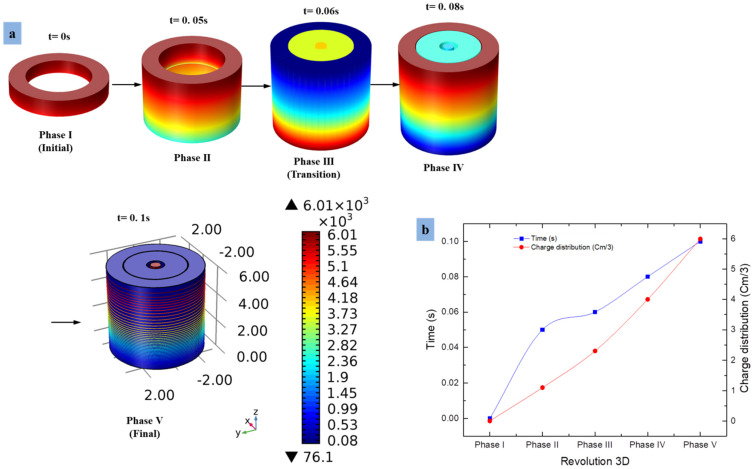
DoD e-jet printing dynamics at different time intervals. (**a**) Comparison of revolution 3D model analysis and electric potential for droplet stability on PET substrate and (**b**) results at different time intervals during the charge distribution.

Revolution 3D model analysis is a method of studying fluid dynamics, which is widely used in the field of micro/nanofabrication. Electric potential is an essential factor that monitors and regulates stability during 3D revolution mode. Similarly, phase I is an inherent property of the phase-field method for cone-jet generation throughout this study. The model corresponds to a specific frequency, damping ratio, and main mode of microdroplet stability. The process for obtaining the corresponding parameters of the model is called revolution analysis. At time interval t = 0 s, the model parameter acquisition methods mainly include zero electric field lines around the combined needle interface. Phase II at time interval t = 0.05 s refers to the method of obtaining specific parametric changes without potential lines around the interface region. When the time increases to t = 0.06 s using specific functional parameters, then transition occurs during the revolution of charge distributions due to high voltages at the straight electrode and combined needle. This phase-change point in phase III is considered critical to the development of the stability of cone-jet morphology. Analysis shows that as the time interval increases more to t = 0.08 s, phase IV, then the obtained input and output parameters of the e-jet printing system remove the electric field lines. For this reason, the voltage is the core of the cone-jet printing technology, which shows the shape of the inner (liquid) and outer (air) layers at different moments when voltage is applied. At the time interval t = 0.08 s, as shown in Figure 6a, the electric field lines disappear for a while due to weak charge density effect. Similarly, when the time period increases to t = 0.1 s during phase V, the molecules of functional liquid expose the combined action of surface tension, viscous shear force, and gravity under high potential of charge distribution. Subsequently, the charge distribution around the combined needle surface is much larger than that of the needle interface. As time passes, the inner liquid accumulates at the nozzle outlet and finally a single hemispherical structure wraps to form cone-jet morphology, as shown in Figure 6a. At present, in the effect of electric field analysis of dynamic behavior of fluid using straight electrodes, charge distribution and time intervals are often directly proportional to each other. However, the post-phase III curve shows zero magnitude due to the transformation analysis of distribution charge density. Thus, the curve in Figure 6b depicts the elimination of the internal stress of the electric potential, and the bond strength to the substrate simplifies the reinforcement process in the subsequent 3D revolution performance test.

### 3.3. Influence of Applied Pulse Voltage on Microdroplet Generation 

**Figure 7:**
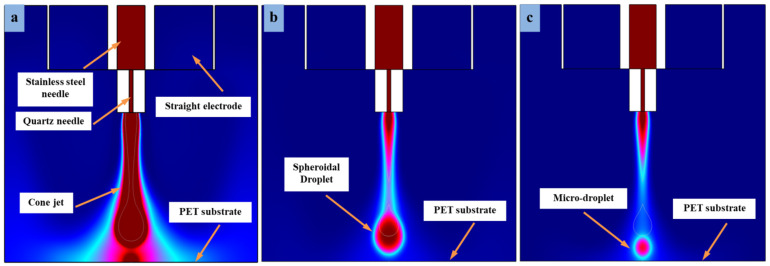
Different-sized microdroplets generated at different applied voltages regulated on combined needle and straight electrode, respectively: (**a**) 7.5 kV, 3.0 kV (**b**) 8.5 kV, 4.0 kV and (**c**) 9.0 kV, 4.5 kV.

The DoD e-jet printing method is considered a reasonable method in flexible printed electronics to generate stable microdroplets, especially on insulating substrates such as PET. In order to control inflation and swelling of microdroplets, optimized voltage values are required throughout the study. In this work, we adjusted the DC pulse voltage on both the combined needle system and the straight electrode. There were a series of experiments performed to obtain the optimized parameters. Figure 7 shows the best simulation results at different pulse voltages, i.e., V_n_ = 7.5 kV, 8.5 kV and 9.0 kV on the combined needle and using different voltage values at the straight electrode, such as V_s_ = 3.0 kV, 4.0 kV, and 4.5 kV, respectively. When the applied voltage was V_n_ = 7.5 kV on the combined needle, then the cone-jet morphology was thick due to low voltage value at the straight electrode (V_s_ = 3.0 kV) and generated abundant residual charges on a PET substrate. Further, as the voltage increased at the combined needle and straight electrode, such as V_n_ = 8.5 kV and V_s_ = 4.0 kV, respectively, then the cone-jet diameter became smaller, leading to the microdripping mode. The simulated droplet size was spheroid due to the strong effect of applied voltage to the straight electrode and much smaller compared to the output at V_n_ = 7.5 kV and V_s_ = 3.0 kV, as shown in Figure 7a,b. The simulation findings, namely those in Figure 7b, demonstrate that the droplet fragments as a result of the combined action of applied voltage and vectors of residual charges that resist their spread across the PET substrate. As a result, it is clear from Figure 7b that the straight electrode effect may faithfully duplicate the droplet-detachment time, one of the key operational components of the phase-field approach.

Subsequently, in another simulation experiment, the voltage was increased to 9.0 kV on the combined needle and 4.5 kV on straight electrodes, respectively. The straight electrodes were positioned parallel to the combined needle at 0.3 mm distances evenly in all the simulation experiments. However, it can be seen in Figure 7c that the droplet diameter is different and very small due to the increase in voltage on the combined needle and straight electrodes. The small-diameter cone jet is generated by the change in electrical potential between the combined needle and straight electrodes without any electric field influence in the experiment. The outflow of a combined needle interface quickly converts the e-jet morphology into microdripping mode. However, this has happened as a result of both the impact of high electrical forces and the influence of poor charge density. Numerical modeling has experimentally demonstrated a connection between the combined effect of a needle system and a straight electrode and the electric field produced by high pulse voltage. On the PET substrate, the diameter of the microdroplets is reduced, but the cone-shape jet vanishes and changes to a microdripping mode. The influence and strength of the electric field were strong towards the surface of flexible PET substrate, although the substrate had considerable nonconductive properties. The cone-jetting phenomenon demonstrates that by controlling high pulse voltage in a straight electrode constructed model, it is possible to print stable microdroplets employing the DoD e-jet printing technology for the application of MEMS devices.

**Figure 8:**
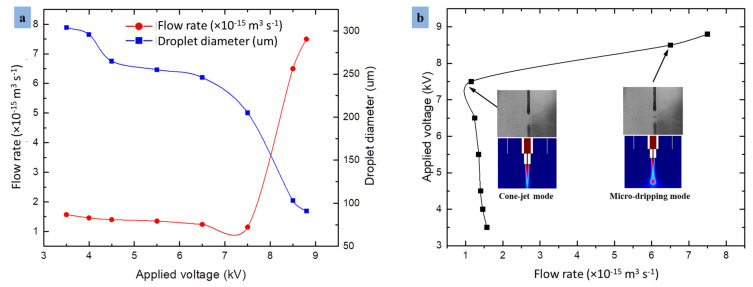
(**a**) Droplet-size dynamic curve at different applied voltages and flow rates, (**b**) comparison of cone-jet formation and microdripping mode.

As shown in Figure 8a, the flow rate is twice the applied voltage, and the two parameters follow a nonlinear relationship corresponding to microdroplet diameter. This relationship indicates that both positive and negative cone-jet behaviors are engaged in droplet formation. The curve shows that if the voltage rises from a certain limit, then the droplet diameter also increases very rapidly due to the excess electric field lines around the needle interface. Similarly, Figure 8b explains the relationship of electric potential and flow rate performed by the combined needle and straight electrode, so that the subsequent microdroplet is of opposite polarity. The optimized values of voltages and flow rate can neutralize the residual charges on the substrate surface for continuous feature during fabrication. The curve displays the cone-jet formation and microdripping mode using single polarity voltage. The approach that uses DC pulse is robust for particle deposition. However, as the voltage fluctuates from the peak value to its optimized value, then no net residual charges accumulate on the substrate surface, regardless of their ability to dissipate the residual charge quickly or not.

### 3.4. Influence of Flow Rate on Microdroplet Generation 

**Figure 9:**
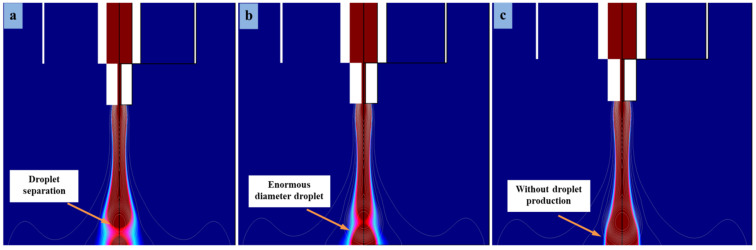
Different-sized microdroplets generated at different applied flow rates: (**a**) 8.6 × 10^−10^ m^3^ s^−1^, (**b**) 3.7 × 10^−8^ m^3^ s^−1^, and (**c**) 4.07 × 10^−8^ m^3^ s^−1^.

Flow rate and DC pulse voltage are two critical parameters to generate a positive influence on the stability of cone-jet morphology and microdroplet diameter. Flow rate is regulated in the functional liquid, which plays an important role in the e-jet printing technology. Hence, a series of simulation experiments were performed by adjusting different flow rate values at the combined needle, such as *f* = 8.6 × 10^−10^ m^3^ s^−1^, 3.7 × 10^−8^ m^3^ s^−1^, 4.07 × 10^−8^ m^3^ s^−1^, 5.6 × 10^−7^ m^3^ s^−1^, and 3.4 × 10^−6^ m^3^ s^−1^. Primarily, a flow rate of *f* = 9.6 × 10^−10^ m^3^ s^−1^ was set in the combined needle system and the functional fluid speed was very low and reached the interface of needle very slowly. Due to this, proper e-jet morphology was not obtained at the needle tip. Thus, by regularly increasing the flow rate from the low value, the e-jet profile evolved and appeared at the needle tip. Finally, when setting the flow rate values from *f* = 9.2 × 10^−10^ m^3^ s^−1^ to 3.4 × 10^−6^ m^3^ s^−1^ then different sizes of cone-jet morphologies were obtained that led to droplet formation, as shown in Figure 9. All entire parameters were kept constant to evaluate the optimized value for generating stable microdroplets on the flexible PET substrate, and were kept constant to estimate the optimized value for the generation of stable microdroplets on the PET substrate. When the flow rate of functional liquid was *f* = 8.6 × 10^−10^ m^3^ s^−1^, then the formation of droplets on the flexible PET substrate was flawless, as it split during microdripping mode, which is shown in Figure 9a. This is due to reduction in the flow rate of functional liquid, which caused the output flow rate to decline to a specific level, resulting in small-droplet formation. Furthermore, as the flow rate increases to *f* = 3.7 × 10^−8^ m^3^ s^−1^ and 4.07 × 10^−8^ m^3^ s^−1^, then cone-jet morphology and droplet diameter increase enormously due to large numbers of liquid particles that accumulate around the needle tip, as shown in Figure 9b,c. This shows that the effect of charge density distribution at the highest flow rate was ordinary around the needle interface. Therefore, the flow rate of the functional liquid was directly proportional to the size of the cone jet and droplet diameter. Moreover, the simulated microdripping mode and controllability of the cone-jet shape were significantly impacted by the ink flow rate.

**Figure 10:**
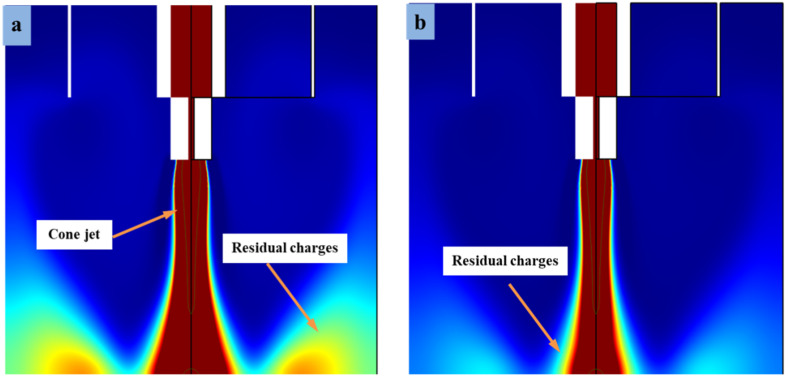
Different-sized microdroplets generated at higher flow rates of (**a**) 5.6 × 10^−7^ m^3^ s^−1^ and (**b**) 3.4 × 10^−6^ m^3^ s^−1^.

Uncertainty in stability and flexibility of the generated cone jet occurred when the ink flow rate increased more than a certain value (*f* = 3.7 × 10^−8^ m^3^ s^−1^) during the simulation experiments. Since the speed of functional fluid can be very high, the effect of electric field generating around the two phases becomes very high. Similarly, this will cause inflation between the needle tip and air gap, which cannot maintain the formation of stable and resolute cone-jet morphology on PET substrates. As we adjusted the highest flow rate (*f* = 5.6 × 10^−7^ m^3^ s^−1^ and *f* = 3.4 × 10^−6^ m^3^ s^−1^), then the Taylor cone profile disappeared due to immense residual charge evolution and the e-jet was taken without the cone angle shown in Figure 10a,b. The simulation experiments determined that the cone-jet was very stable at the low flow rate (*f* = 8.6 × 10^−10^ m^3^ s^−1^), which is suitable for printing various microstructures on PET substrates. Consequently, to look for optimal parameters, all simulation results that affected the cone-jet morphology were evaluated, and the droplet was stable during microdripping mode on the substrate surface. In the end, the existing simulation model determined that the high pulse voltage value of V_n_ = 9.0 kV and V_s_ = 4.5 kV on the combined needle and straight electrode and low flow rate (*f* = 8.6 × 10^−10^ m^3^ s^−1^) were the optimal parameters. Thus, these parameters are fully capable of printing microdroplets on flexible insulating substrate for the application of flexible electronic systems.

### 3.5. Influence of Needle–Substrate Distance on Cone-Jet Shape

**Figure 11:**
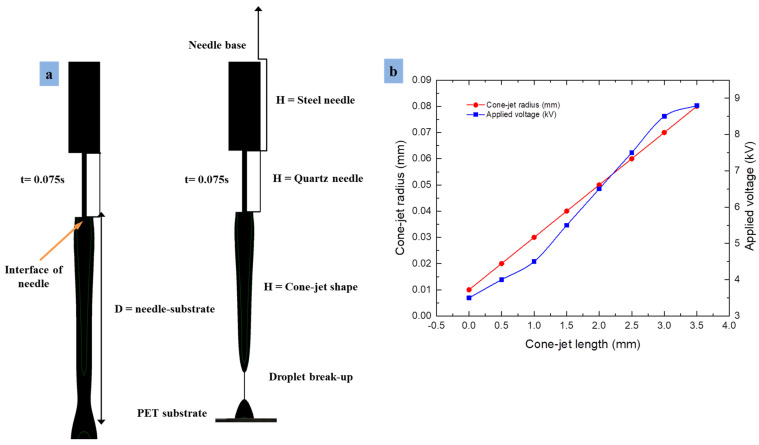
Formation of cone jet and microdroplets at different distances between needle tip to substrate: (**a**) 240 μm and 200 μm; (**b**) relationship between cone-jet radius and cone-jet length.

The effect of needle–substrate distance was investigated on cone-jet formation by keeping the flow rate and applied voltage (i.e., *f* = 8.6 × 10^−10^ m^3^ s^−1^, V_n_ = 9.0 kV, V_s_ = 4.5 kV) constant during the simulation. A series of experiments were performed by adjusting distances between the needle tip and substrate, hence the best results (i.e., 240 μm and 200 μm) are discussed in which the small cone jet was formed and on the other hand the droplet was split on substrate surface. As can be seen from Figure 11a, as the distance between the combined needles to substrate reduces, the Taylor cone becomes smaller near the interface and then over time cone-jet width shrinks. It was determined that the cone-jet size was directly proportional to the needle–substrate distance. This happens mainly because reducing the distance increases the electric field strength but reduces diameter of the cone jet. As a result, the tangential electric field force acting on the cone jet becomes small. The simulation experiments showed that when we reduced the distance to 200 μm, the cone-jet mode transformed into microdripping mode. Similarly, Figure 11a shows two results of simulation experiments at the same time intervals (t = 0.075 s) demonstrating cone-jet profile formation and droplet generation.

During the simulation, the shape of cone jet showed that the electric field force generated when the printing distance was too high, not sufficient to overcome the surface tension with the viscous force. Thus, the liquid accumulated at the outlet of the combined needle was larger than the liquid sprayed on the substrate, which led to the coarse cone-jet morphology. Therefore, the lower the print distance, the greater the tangential electric field force and the smaller the cone-jet diameter. However, simulation results indicate that when the printing distance is less than 200 μm, then liquid sprayed to the substrate is greater than the liquid that the syringe pump can supply. This particular finding shows that the liquid will break the needle to form a cavity. Moreover, it displays the effect of electric potential intensity on cone-jet length, and as the needle–substrate distance increases, and then cone-jet length will routinely increase with large diameter of the Taylor cone profile. Similarly, Figure 11b shows the relationship between cone-jet radius and cone-jet length keeping applied parameters constant. The cone-jet morphology directs the e-jet towards microdripping mode at high voltage potential, which causes the formation of microdroplets on the flexible PET substrate surface.

### 3.6. Experimental Study under Optimized Simulation Results

#### Printing of Droplets on Flexible Substrate

**Figure 12:**
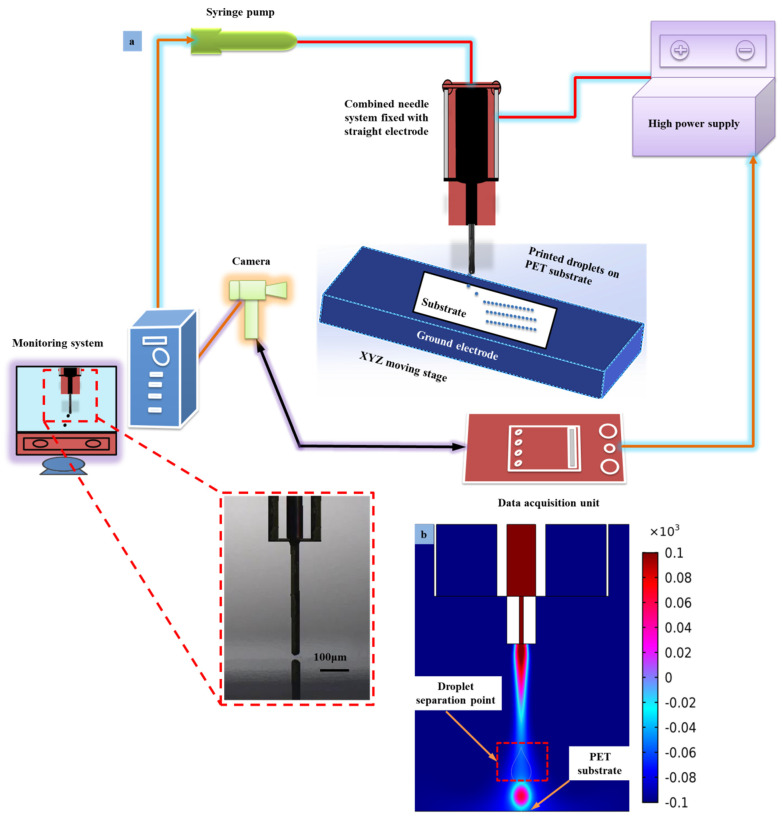
(**a**) Schematic of DoD e-jet printing process equipped with the straight electrode needle system, (**b**) formation of microdroplets during numerical simulation.

Figure 12 illustrates the specific cone-jet morphology obtained under the optimized parameters. A series of experimental investigations were carried out using optimized parameters to verify the simulation results. The findings show that the real cone-jet and droplet diameter are inversely related to applied voltage, but the distance between the needle and the substrate and the liquid flow rate are proportionate. The size of the printed microstructures likewise complies with this requirement, demonstrating the accuracy of the simulation model and its output. The simulation findings show that a higher applied pulse voltage is required to produce a small-diameter cone-jet morphology and smaller printed microstructures. Similarly, as mentioned in the numerical simulation, the straight electrode design is very effective and innovative. Furthermore, the similar straight electrode diameter was used during the experimental study. Due to the utilization of an insulating PMMA holder, the high pulse voltage does not lead to the opposite effect of eddy current on the outside surface of the straight electrode. Therefore, the goal of this study was to evaluate the simulation by printing stable microstructures on flexible surfaces using UF3808 as a functional paint. The process parameters of voltage, flow rate, and needle–substrate distance integrate DoD e-jet printing with flexible insulating substrates. The schematic of the DoD e-jet printing process equipped with the straight electrode needle system is shown in Figure 12a, and similarly the droplet generation on the flexible PET substrate during numerical simulation is shown in Figure 12b. The minimum fluid flow rate and the applied pulse voltage (*f* = 8.6 × 10^−10^ m^3^s^−1^, V_n_ = 9.0 kV, V_s_ = 4.5 kV) were used as optimized parameters to print various microstructures using constant frequency of 100 Hz and duty cycle of 80%. Subsequently, various DoD patterns began to print directly on the flexible PET substrate.

**Figure 13:**
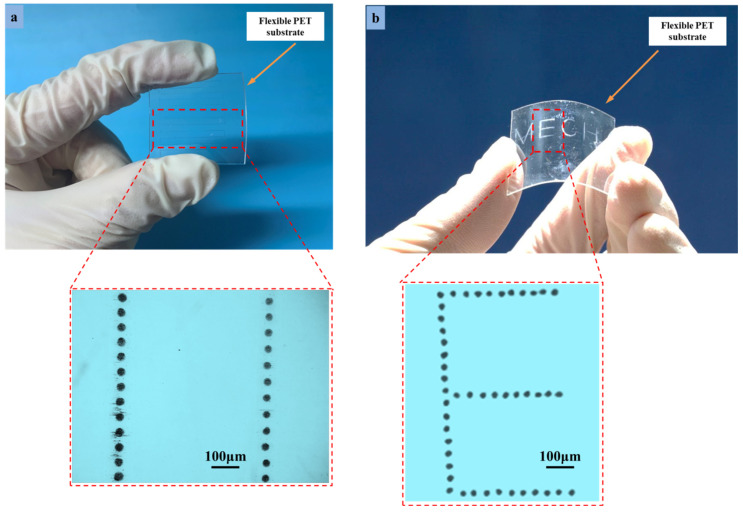
The results of DoD e-jet printing of microstructures and patterns on PET flexible substrates: (**a**) linear droplet array (**b**) flexible “E” pattern in MECH microstructures.

Figure 13 illustrates different microstructures that are printed on the flexible PET substrate having a thickness of 0.5 mm. The parameters of the optimized process are the same as those employed in the numerical simulation. In the printing process, variables including the printing trajectory, printing speed, applied voltage, and flow rate are connected and managed in accordance with the outcomes of simulations. Using UF3808 with a viscosity of 450,000 cps, the “droplet string” and flexible “E” pattern in MECH microstructures were printed at room temperature in Figure 13a,b. There is no breakdown or buildup of glue lines, and the microstructures are continuous and homogenous. The aspect ratio of the printed microstructures is around 0.4, and the smallest droplet was 12.5 μm. The microstructure printing outcomes show that the settings used are ideal for flexible printed electronics to improve their stability and resolution when printed using the DoD e-jet printing method. Additionally, the simulation model demonstrated the DoD method’s competence and adaptability for well-adjusted microscale structures on flexible PET substrates.

The distance between needle and substrate was kept at 350 μm, which was not aimed at producing a microdrip regime due to the strong repulsive force integrations on the flexible substrate. In parallel, at a distance of 350 μm from needle tip to substrate during the simulation, the droplet was stable and reduced the effect of residue charges. The minimum droplet size was measured to be 12.5 μm within the printed microstructures deprived of coffee-ring effect. Similarly, the actual droplet diameter was measured to be 13 μm on the PET flexible substrate, which realized the standard deviation in the data set that was measured at 0.5 μm. This is due to the influence of the variation in surface energy of the polymer substrate. Therefore, a minimum needle–substrate distance of 200 μm was preferred to print stable microstructures on a flexible substrate.

## 4. Conclusions

In this article, we propose a phase-field approach to produce a stable cone-jet profile that can be employed to fabricate micro- and nanoscale structures on flexible insulating substrates. This work provides an original and cutting-edge needle–straight electrode combination that significantly affects the stability of microdroplets. The numerical simulation of cone-jet creation was carried out as the first step in this work. For Taylor cone creation, a force model was created, and the effects of each force on the cone jet were examined. The Navier–Stokes equation and the Maxwell pressure tensor approach were used to obtain the motion equation, electric field equation, and interface tracking equation between fluids. Boundary conditions were established once the physical model was converted into a symmetrical model. To examine how the important printing factors affected the cone jet, a numerical simulation of the e-jet was conducted. Additionally, the effect of the needle and straight electrode voltage on cone-jet morphology was investigated. The influence of the electric potential was also studied in 2D and 3D plot assembly to investigate the inflation on cone-jet morphology. The results demonstrate that the cone-jet and droplet diameters are inversely proportional to the voltage and are proportional to the needle–substrate distance and the liquid flow rate. Lastly, optimized simulation parameters were used to print stable microstructures on flexible PET substrate by using UF 3808 ink in experiments. The findings of the experiments revealed the simulation model’s effectiveness in printing microstructures on stretchable insulating surfaces and in establishing a feasible route for manufacturing MEMS devices. This model is unique, inexpensive, quick, and ecologically friendly in its fabrication process.

## Figures and Tables

**Table 1:** Physical properties of functional ink used during the simulation and experiments.

Properties	Values
Dynamic viscosity	360 mPa·s
Specific gravity	1.16
Shelf life	365 days
Surface tension	0.031 N/m
Dielectric constant	3.24
Storage modulus	260 N/mm^2^
Dissipation factor	0.0088

**Table 2:** Simulation boundary conditions of electrostatic field and flow field.

Boundary	Electrostatic Condition	Hydrodynamic Condition
A: Needle inlet	$n\cdot \vec{j}$= 0	*u* = Q_inner_/A_inner_
B: Needle wall	V= *V*_0_	w = 0, *u* = 0
C: Straight electrode wall	V= *V*_0_	w = 0, *u* = 0
D: Straight electrode charge	V= *V*_0_	w = 0, *u* = 0
E: Needle outlet	$n\cdot \vec{j}$= 0	u = Q_outer_/A_outer_
G: Axi-symmetry	$\frac{dV}{dr}$= 0	$u=0,\;\frac{dw}{dr}$ = 0
F: Boundary of computational territory	V= 0	*p* = 0, *u* = 0
H: Air territory	V= 0	*p* = 0, *u* = 0

## Data Availability

Not applicable.
